# Leveraging CRISPR Cas9 RNPs and Cre-*loxP* in *Picochlorum celeri* for generation of field deployable strains and selection marker recycling

**DOI:** 10.3389/fmicb.2025.1588625

**Published:** 2025-07-14

**Authors:** Tyson A. Burch, Anagha Krishnan, Matthew C. Posewitz

**Affiliations:** ^1^Department of Chemistry, Colorado School of Mines, Golden, CO, United States; ^2^National Renewable Energy Laboratory, Department of Energy, Golden, CO, United States

**Keywords:** picochlorum, Cas9, Cre-LoxP, CRoxP, algae, LHC, depigmentation, biomass

## Abstract

As new highly productive strains of algae are discovered and developed to meet the energy, chemical, and food requirements of the future, genetic engineering of those strains in a manner that yields deployable transformants is paramount. This study introduces the novel CRoxP (Cas9 RNPs coupled with an inducible CRe-loxP) system for rapid generation of marker- and transgene-free strains of *Picochlorum celeri*. The CRoxP system allows reuse of selection markers without Cas9 expression *in vivo*, eliminating many of the bottlenecks associated with conventional CRISPR Cas9 use for precise genome editing. In *P. celeri*, transformants were generated with a turnaround time as short as 21 days between transformation and being ready for another round of transformation with the same selection marker by using the CRoxP system. As a use-case for CRoxP, depigmented strains of *P. celeri* were generated by multiplexed Cas9 disruption of major *LHCII* genes followed by either a second round of LHCII targeting, or knockout of an *LHCI* gene. One transformant tested in flask culture (R6) exhibited similar biomass production to the wild type with 46% less Chl *a* + *b* on a biomass basis. In photobioreactors and under diel light simulating a solar day, a transformant (LhcBM31) exhibited 34 g AFDW m^–2^ d^–1^ with 54% less Chl *a* + *b* on a biomass basis vs. wild type.

## 1 Introduction

As the global population expands and the demand for environmentally friendly sources of food, materials, and fuel increases, novel and sustainable sources of these fundamental resources must be developed. The promise of microalgae to fill some of these needs has been discussed for decades because of their ability to grow rapidly, use land that is unsuitable for growing crops, produce a variety of products, and be used for bioremediation (Benemann, 1997; Richmond and Zou, 1999; Chisti, 2007; Becker, 2007; Pulz and Gross, 2004; Muñoz et al., 2009). With some notable exceptions, the promises of microalgae have remained unfulfilled for various reasons, partly due to the limitations of the most commonly studied strains that either did not perform well in outdoor ponds and/or the genetic tools to engineer new strains did not exist. With the advent of CRISPR (Clustered Regularly Interspaced Short Palindromic Repeats) Cas9 genome editing (Doudna and Charpentier, 2014), the decreased cost to read and write DNA, and the discovery of highly productive and robust algal chassis strains, substantial progress toward the deployment of algae for mass production of products is advancing.

A key focus of contemporary research is identifying and validating highly productive strains that have the innate ability to produce valuable products and/or can serve as a chassis that can be further engineered (Krishnan et al., 2025). Building the genetic tools to engineer these new strains is of utmost importance to study their metabolic underpinnings, to fine tune their attributes, and to incorporate novel capabilities. This study builds upon the work done by Krishnan et al. (2020) in the fast-growing marine green alga *Picochlorum celeri*, where CRISPR Cas9 RNPs (ribonucleoproteins) were assembled *in vitro* and used to produce knockout transformants. By combining Cas9 RNPs for precise genome manipulation with selection marker inserts equipped with an inducible Cre-*loxP* system to remove both the selection marker and Cre recombinase simultaneously, rapid generation of transformants that lack any heterologous genes or selection markers is possible. To simplify discussion of this system, genome editing using externally synthesized Cas9 RNPs coupled with an inducible Cre-loxP system will be referred to as a CRoxP system throughout the text.

There are a variety of methods in the literature for deploying CRISPR-Cas9 in algae including constitutively expressing Cas9 (Verruto et al., 2018; Vogler et al., 2021), using plasmids to transiently express Cas9 (Jiang et al., 2014; Wang et al., 2016), and using externally synthesized RNPs (Krishnan et al., 2020; Badis et al., 2021). CRISPR-Cas9 was also successfully used for markerless, precise and rapid genome editing in cyanobacteria (Cengic et al., 2022). Mosey et al. (2021) present a review of various methods to apply electrophoresis transformation techniques to other non-model algal species, which could likely be applied to use Cas9 RNPs for targeted genome engineering in those strains.

While constitutive expression simplifies transformant generation after the establishment of a good chassis and eliminates the cost of the Cas9 protein, there are concerns that over time, constitutive expression of Cas9 could lead to off-target editing that could accumulate undesired mutations. Both constitutive and transient expression of Cas9 in the target cells requires proper expression and folding of the Cas9 protein, which could be difficult in some strains. While the cost per transformation of Cas9 using *in vitro*-synthesized RNPs is higher, it eliminates the concerns of continuous expression accumulating unintended mutations and does not require proper expression and folding of the Cas9 protein in the target cells. Kim et al. (2014) showed that RNP delivery in human cells decreases off-target effects compared to constitutive Cas9 expression, and Shin et al. (2016) came to similar conclusions for the model alga *Chlamydomonas reinhardtii*.

Cre-*loxP* is a widely used system to excise, rearrange, and control the expression of DNA *in vivo* in a variety of organisms (Sauer, 1987; Abuin and Bradley, 1996; Zuo et al., 2001). Cre recombinase locates and causes DNA recombination at *loxP* sites, which if oriented in a specific way, can be used to remove the DNA between the two *loxP* sites. The DNA between two *loxP* sites is termed floxed or flanked by *loxP* sites. Editing by Cre-*loxP* leaves behind, at a minimum, a *loxP* scar since the two *loxP* sites involved are spliced together in the middle to yield a single *loxP* site. There are several examples of Cre-*loxP* use in algae, mainly as a method for targeted insertion of DNA into *loxP* sites of a strain (Heitzer and Zschoernig, 2007; Kasai and Harayama, 2016). Verruto et al. (2018) used Cas9 coupled with Cre-*loxP* in algae to generate transformants in *Nannochloropsis gaditana* that allowed recycling of the selection marker and multiple rounds of gene knockouts in a constitutive Cas9 expression strain.

Because microalgae have a high chance of escape from containment and could pass genetic material to other comingled bacteria and eukaryotic single-celled organisms in a pond, genetically modified microalgae face significant regulatory hurdles in their outdoor deployment (Spicer and Molnar, 2018; Shokravi et al., 2021). Therefore, testing GMO algae with intact antibiotic resistance markers in their genome introduced during genetic engineering is currently restricted in outdoor ponds without regulatory approval. Deployment of GMO algae with other kinds of heterologous gene expression currently requires approval of the genes by the Environmental Protection Agency (EPA) in the United States. The CRoxP system described here to generate gene knockout transformants yields strains that lack selection markers and other heterologous genes, so they are more readily deployable in outdoor ponds by current regulatory guidelines. Using the CRoxP system to generate transformants for overexpression of native genes using native promoters and terminators would also be readily deployable under current regulations. Finally, the CRoxP system could be used to express heterologous genes without any selection markers or other genes in the resulting strains, simplifying the regulatory approval process to the specific heterologous genes themselves.

Because many organisms only have a few selection markers available for transformations, the ability to recycle selection markers for multiple rounds of genetic modification to stack alterations in a single strain is required for testing some hypotheses and/or for generating specialized strains. The CRoxP system enables many rounds of genetic modification using the same selection marker.

A common target for strain improvement in algae is pigment reduction to improve light use efficiency in dense algal ponds. The theory is that by reducing the number of photons absorbed by the cells on the surface of a pond, more light can penetrate to cells deeper in the culture, making them productive. In addition, by reducing the number of photons absorbed by cells on the surface, it is expected that less light will be wasted by those cells through non-photochemical quenching pathways, thus improving light-to-biomass conversion efficiencies. See Krishnan et al. (2023) for a review of the theory behind depigmentation and references to other works in this area of study. Also see Verruto et al. (2018) for another example of using a constitutively expressed Cas9 system coupled with Cre-*loxP* for selection marker-free, but not heterologous gene-free depigmented transformants of *N. gaditana*. As an example use-case for the CRoxP system, Light Harvesting Complex (LHC) genes were targeted for disruption in *P. celeri* to generate depigmented strains in the present study. These genes represent a good use case because there are putatively 5 different *LHCBM* and 9 different *LHCA* genes in *P. celeri*, as determined by comparative protein BLASTs with *Chlamydomonas reinhardtii* genes, so iterative rounds of transformations are required to target multiple and different LHC combinations for knockout.

## 2 Materials and methods

### 2.1 Strain and culture maintenance

*Picochlorum celeri* TG2 from Weissman et al. (2018) was cryopreserved and revived periodically using the methods described in Elliott et al. (2012) to preserve the traits of the originally isolated strain without laboratory phenotypic drift. After revival in dense liquid culture medium (see media formulations in section 2.2) cultures were streaked onto QATM agar plates with certain modifications as needed and moved to liquid culture medium as needed (Krishnan et al., 2021).

### 2.2 Media formulations

#### 2.2.1 QATM medium

QATM media contained either 4.4 mM urea, 8.8 mM sodium nitrate (NaNO_3_), or 8.8 mM ammonium bicarbonate (NH_4_HCO_3_), 25% filtered seawater, 434.4 μM NaH_2_PO_4_⋅H_2_O, 0.234 μM FeCl_3_⋅6H_2_O, 0.234 μM Na_2_EDTA⋅2H_2_O, 0.786 nM CuSO_4_⋅5H_2_O, 0.52 nM Na_2_MoO_4_⋅2H_2_O, 1.53 nM ZnSO_4_⋅7H_2_O, 0.84 nM CoCl_2_⋅6H_2_O, 1.82 nM MnCl_2_⋅4H_2_O, 2.96 μM thiamine HCl, 20.5 nM biotin, 3.7 nM cyanocobalamin and 20 mM MOPS (pH 7.6). For solid agar plates, 1% BD Bacto Dehydrated Agar was added, and 0.4% low-melt agarose (VWR) was used for overlay plating. 80 μg mL^–1^ nourseothricin (CNAT), and 20 μg mL^–1^ phleomycin were used in plate and overlay agar as appropriate.

#### 2.2.2 Dense medium

An Instant Ocean-based medium referred to as Dense Marine medium was used for experiments where high culture densities were desired. See Weissman et al. (2018) and LaPanse et al. (2024) for the medium formulation.

### 2.3 Plasmid construction

The transformation plasmids used in this study were constructed using a printed Cre-*loxP* cassette from Twist Bioscience (CA, United States). The Cre recombinase sequence and intron placement were taken from the XVE Cre construct of Zuo et al. (2001) GenBank accession number AF330636. The *P. celeri* promoters and terminators were identified using the published genome (accession number PRJNA598876) from Becker et al. (2020) and amplified using Q5 hot-start 2x master-mix (New England Biosciences, MA, United States). Plasmid components were assembled using 2X Gibson Assembly master-mix using standard protocols and cloned into 10-beta competent cells (New England Biosciences, MA, United States). The putative *P. celeri* endoglucanase intron was added to the Cre recombinase gene to prevent expression in *E. coli* causing recombinase activity during plasmid generation. Where appropriate, two-step Gibson Assembly was performed to avoid assembly of pieces with *loxP* sites at the ends because the homology between them prevented proper assembly. The full sequences and plasmid maps of both the CNAT (pNRCreCNAT) and phleomycin (pNRCreBle) plasmids are provided in the Supplementary materials and both plasmids were submitted to Addgene and made publicly available. Primer sequences used for plasmid construction as well as PCR analysis of gene editing events are provided in the Supplementary materials.

### 2.4 Transformation of *P. celeri* with Cas9 RNPs and inducible Cre-loxP construct

Transformation of *P. celeri* was performed as described in Krishnan et al. (2020, 2023) with several differences. First, 8 μg of cut plasmid was used to compensate for the larger size of the selection marker with inducible Cre recombinase cassette. It was found that a concentration greater than 1.5 μg μL^–1^ was necessary for the cut plasmid to yield high numbers of transformants (50 +) with an optimal concentration of 2 μg μL^–1^ (data not shown). Second, two-fold sgRNA (100 pmol) to Cas9 protein (50 pmol) was used to generate the Cas9 RNP. Finally, recovery following electroporation was carried out in 5 mL sterile tubes that allowed gas exchange with the atmosphere in a 33°C incubator with 1% CO_2_, incubated for 3 h then lightly vortexed and incubated another 3 h prior to plating on selection plates using top agar. Transformation efficiency with Cas9 RNPs was similar to transformations without Cas9 RNPs that rely on random selection marker insertion into the genome, yielding 100+ transformants per transformation. The selection marker was located in the Cas9 RNP targeted cut site for all strains analyzed.

### 2.5 Cre-*loxP* activation for marker removal

Candidate antibiotic resistant colonies were patched onto QATM urea plates containing 80 μg mL^–1^ CNAT. Cre-*loxP* activation was carried out either by streaking to single colonies on QATM- NaNO_3_ plates without antibiotics or by resuspending in QATM- NaNO_3_ liquid media without antibiotics (20 mL culture volume in a 50 mL flask). For the liquid induction strategy, culture growth was maintained by removing 18 mL of culture and adding 18 mL fresh medium every 2 days. To obtain clonal isolates, a sterile loop was dipped into the liquid and struck to single colonies on QATM NaNO_3_ plates without antibiotics. After initial testing, the liquid induction strategy with 3 days of induction was used for generating depigmented transformants.

### 2.6 Flow cytometry

Samples were diluted 1:20 with fresh medium and then measured on an Attune NxT acoustic focusing flow cytometer (ThermoFisher Scientific) using blue laser and detectors to measure forward scatter and chlorophyll fluorescence (695/40).

### 2.7 Chlorophyll quantification

A culture sample (from 0.1 to 0.5 mL depending on culture density) was put into 1.7 mL microcentrifuge tubes, centrifuged at 8,000 g for 5 min, the supernatant was removed, 1 mL ice cold MeOH was added to the pellet and pipetted to break up the pellet, then quickly put on ice in the dark to incubate. After 10 min, the tubes were vortexed for 3 s and placed back on ice. After another 10 min, the tubes were vortexed again and added to a prechilled (4°C) centrifuge and centrifuged for 5 min at max speed. The 0.7 mL of supernatant was carefully moved to a cuvette and the absorbance was read at 652, 665, and 750 nm using a P4 UV-Visible Spectrophotometer VWR. Chlorophyll concentration was calculated using the equations of Ritchie, 2006.

### 2.8 Whole-cell absorption spectra

Culture samples were diluted 1–5 with fresh medium in quartz cuvettes and the absorbance spectrum was taken from 400 to 750 nm using a P4 UV-Visible Spectrophotometer VWR spectrophotometer. The resulting chromatograms were normalized to 440 nm.

### 2.9 AFDW measurement

Five to ten milliliter samples were transferred to 15 mL falcon tubes depending on culture density to avoid clogging the pre-ashed filters. Otherwise, the protocol used was the same as Burch et al. (2024).

### 2.10 77K chl fluorescence

Harvested samples were diluted to 5 μg mL^–1^ chlorophyll using fresh dense medium and then stored in the dark for 10 min and pulled into a 2 mL plastic pipette without gradations and flash frozen in liquid nitrogen. The frozen samples were transferred to a liquid nitrogen dewer for fluorescence measurement in a Fluorolog instrument using 440 nm excitation light and measuring from 640 to 820 nm emission light in 1 nm increments. The instrument was set to 1 nm slit width, and readings were repeated 32 times and averaged for each sample. The resulting chromatograms were normalized to 702 nm. Biological replicate samples were averaged after normalization to 702 nm.

### 2.11 Flask experiments

Cells were taken from patch plates and resuspended in dense medium with urea as the nitrogen source. 20 mL cultures in 50 mL flasks were grown in an incubator set at 33°C, 1% CO_2_, 800 μmol photons m^–2^ s^–1^, ∼120 RPM on an orbital shaker table, for 1 day to acclimate the cells to liquid culture. After acclimation, culture density was set to an OD750 of 0.1 in fresh flasks using fresh medium and grown for 48 h. Samples were taken after 48 h for analysis.

### 2.12 ALGiSIM experiments

Cultures were grown in photobioreactors as described by Cano et al. (2021) and Krishnan et al. (2023) with insolation simulating a diel light curve for Mesa AZ in mid-May with 2,200 μmol photons m^–2^ s^–1^ at the peak of the day. Constant 33°C temperature, agitation with magnetic stir bars spinning at ∼125 RPM, and bubbling with 2.25% CO_2_ mixed with air at a flow rate of 400 mL/min were maintained throughout the experiment. Cultures were automatically sampled and then 50% of the culture was removed and replaced with fresh medium once per day, 1 h before sunset.

## 3 Results

### 3.1 Development of inducible Cre-*loxP* system in *P. celeri*

**FIGURE 1:**
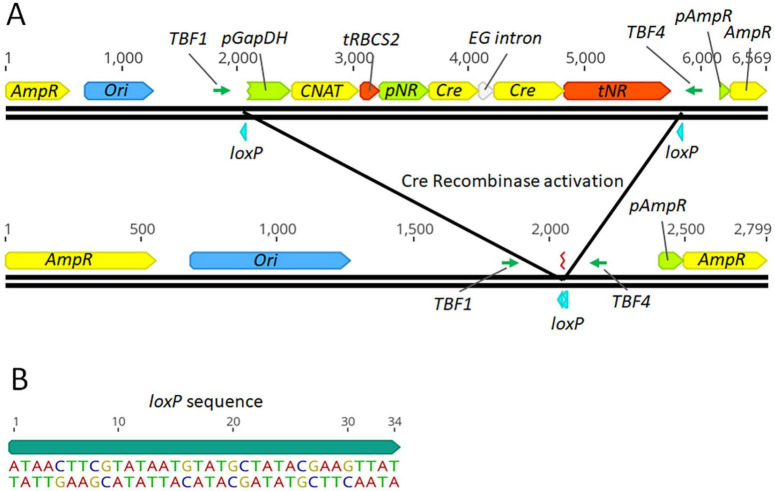
Map of the linearized *P. celeri* transformation plasmid and resulting DNA after successful Cre based recombination **(A)** and the *loxP* sequence used **(B)**. The transformation construct has a CNAT selection cassette, nitrate inducible Cre-recombinase cassette, and *loxP* sites flanking the selection marker and inducible Cre recombinase. The selection marker is promoted and terminated by the native *P. celeri GapDH* and *RBCS2* regions, respectively. The nitrate-inducible Cre-recombinase gene contains a putative endoglucanase intron from *P. celeri* to prevent expression in *E. coli* during plasmid preparation. The construct was cut in the ampicillin resistance gene to prevent its expression upon introduction into *P. celeri*. TBF1 and TBF4 were the primers used to analyze Cre-recombinase activity by PCR. Below the linearized transformation plasmid is a map of the resulting DNA sequence after successful Cre recombinase activity, leaving behind a single *loxP* site and the gene-free plasmid backbone.

To assess the functionality of the Cre-*loxP* system in *P. celeri*, an initial transformant with the inducible Cre-*loxP* system was randomly inserted into the *P. celeri* genome (without Cas9). A map of the linearized nitrate-inducible Cre-*loxP* system and the resulting DNA insert after successful Cre recombinase activity are shown in Figure 1A. The Cre recombinase gene was expressed under the control of the native *P. celeri* nitrate reductase promoter and terminator. In this construct the CNAT resistance cassette and the inducible Cre recombinase cassette were flanked by *loxP* sites oriented in the same direction. Figure 1B shows the *loxP* sequence and direction where the 13 bp on either end are reverse complements of each other and the central 8 bp impart directionality to the *loxP* motif. This initial random-insertion transformant was analyzed through a series of tests to probe the functionality of the Cre-*loxP* system, the inducibility of the nitrate reductase promoter/terminator regulatory elements (see section 3.1.1), and to assess the best method for activation of the Cre-*loxP* system to obtain marker- and Cre-less transformants (see section 3.1.2). It was found that the Cre-*loxP* construct exhibited similar transformation efficiencies to a construct with only a selection marker present, suggesting the larger construct, and presence of the inducible Cre-recombinase, did not reduce transformation efficiency (data not shown).

#### 3.1.1 Analysis of *P. celeri* nitrate reductase promoter/terminator for inducible regulation of Cre recombinase expression

**FIGURE 2:**
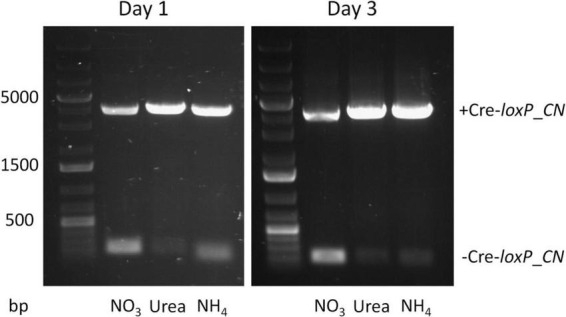
Electrophoresis gel showing PCR results for the Cre-*loxP* strain generated by random insertion into the genome after 1 (left) and 3 (right) days of growth in QATM liquid medium with either NO_3_^–^, urea, or NH_4_^+^ as the nitrogen source. Expected band size for “no Cre recombinase” activity was 4,051 bp (+ Cre-*loxP_CN*) and the expected band size when Cre recombinase successfully excised the selection marker and inducible Cre cassette was 181 bp (-Cre-*loxP_CN*). The faint small bands for both urea and NH_4_^+^ indicate some level of leakiness of the nitrate reductase promoter/terminator.

Currently no information is available on inducible promoters/terminators in *P. celeri*, therefore, selection of candidates relied on promoters/terminators shown to be inducible in other organisms. The nitrate reductase promoter/terminator was shown to be inducible by nitrate in a variety of other organisms so it was selected as a candidate in *P. celeri*. The XVE (estrogen-based) inducible system in plants (Zuo et al., 2000) was attempted for use in *P. celeri* but was non-functional over a range of estrogen additions to the culture medium (data not shown). To assess the inducibility of Cre- recombinase controlled by the native nitrate reductase promoter and terminator of *P. celeri*, the non-targeted transformant described above was transferred to QATM liquid culture media without antibiotics with NO_3_^–^, urea, or NH_4_^+^, respectively, as the nitrogen source and grown as described above. Samples were taken after 1 and 3 days for PCR analysis to determine whether Cre recombinase was expressed and active in the presence of any of the three nitrogen sources. Figure 2 shows electrophoresis gel images of the resulting PCR products for PCR reactions that bridged the *loxP* sites of the Cre-*loxP* system between the TBF1 and TBF4 primers shown in Figure 1A. Expected band sizes were 4,051 bp when the selection marker and inducible Cre recombinase cassettes were still present (+ Cre-*loxP_CN*) and 181 bp when Cre recombinase had successfully excised the DNA between the *loxP* sites (-Cre-*loxP_CN*). The stronger 181 bp band for NO_3_^–^ than for both urea and NH_4_^+^ indicated that the nitrate reductase promoter and terminator were functioning as an inducible promoter/terminator pair. Successful activity of Cre was seen after 1 day of induction by NO_3_^–^. There are faint bands for both urea and NH_4_^+^, indicating that there was at least some leakiness in the inducible promoter, though it was not possible to deduce the extent based on PCR evidence. More in depth study of inducible promoters in *P. celeri* would be beneficial to its development as a bioproducts chassis strain but for the purpose of this study, the results shown in Figure 2 verified that NO_3_^–^ does induce Cre expression and the use of either urea or NH_4_^+^ was suitable for transformations where the activity of Cre recombinase needed to be minimized. For the remainder of experiments and transformations, urea was used as the non-induction nitrogen source.

#### 3.1.2 Comparison of Cre-*loxP* induction techniques for simultaneous marker and Cre recombinase cassette removal

**FIGURE 3:**
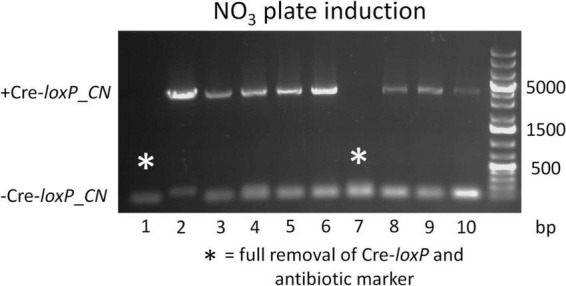
Electrophoresis gel image of a culture struck directly to single colonies on an QATM NO_3_^–^ induction plate, grown to colonies large enough to pick and patch, then tested by PCR for selection marker and inducible Cre recombinase cassette removal. Single small bands of 181 bp without a larger band indicated successful Cre-*loxP* activity without cells present that had not undergone Cre recombination, whereas a small and large (4,051 bp) band indicated that Cre activity likely occurred after the single cells stage and had only occurred in a portion of the cells.

**FIGURE 4:**
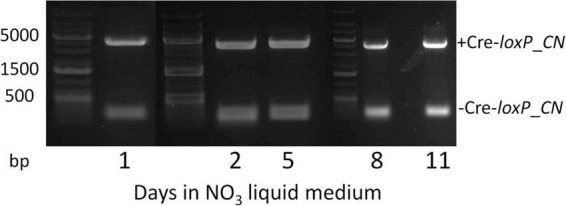
Electrophoresis gel image of a culture grown in QATM NO_3_^–^ induction liquid medium, sampled after 1–11 days of growth with regular dilutions, and tested by PCR for Cre activity. The small 181 bp bands indicate successful Cre activity.

To determine an efficient method to induce Cre recombinase and generate transformants free of a selection marker and inducible Cre recombinase cassette, direct plating to single colonies on induction plates (QATM NO_3_^–^ plates without antibiotics) and growth in liquid induction medium (QATM NO_3_^–^ liquid medium without antibiotics) followed by streaking to single colonies on induction plates were compared. Figure 3 shows an electrophoresis gel image of single colonies tested for selection marker and Cre recombinase cassette removal in cells directly plated on induction plates. Plates were grown until colonies could be picked, patched, and analyzed by PCR. This process took between 8 and 10 days from when the cells were struck on induction plates and resulted in 20% of the colonies tested showing successful removal of the selection marker and inducible Cre recombinase cassette, indicated by stars in the figure. Figure 4 shows an electrophoresis gel image visualizing PCR products amplified from a liquid induction culture at different time points over 11 days.

**FIGURE 5:**
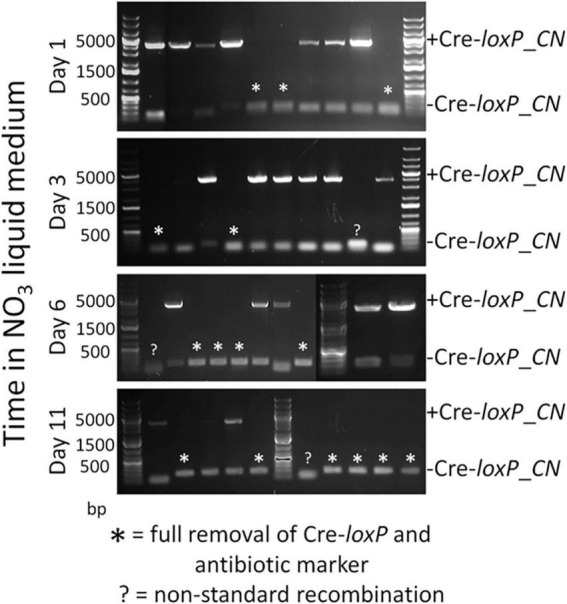
Electrophoresis gel images of PCR analyses of the culture in Figure 4 struck to single colonies on QATM NO_3_^–^ induction plates after 1–11 days of growth in QATM NO_3_^–^ induction liquid medium as indicated on the *Y*-axis. Full removal of the selection marker and inducible Cre recombinase cassette is indicated by stars above the smaller bands at 181 bp while partial removal is indicated by the presence of the larger band at 4,051 bp. Several isolates displayed unique band sizes that may indicate non-standard recombination events among other possibilities.

To analyze the proportion of cells in which Cre recombinase had successfully removed the selection marker and Cre recombinase cassette, the liquid culture from Figure 4 was plated to single colonies on QATM NO_3_^–^ induction plates, grown until colonies could be picked and patched, then the patches were analyzed for complete removal of the selection marker and inducible Cre recombinase cassette. The resulting PCR analyses are shown in the electrophoresis gel images in Figure 5. After 1 day in liquid induction medium followed by streaking to singles on induction plates, 30% of colonies tested showed successful marker removal and this success rate increased to 60% by day 11. These results indicate that induction of Cre activity by direct streaking to single colonies on induction plates or induction in liquid media followed by streaking to single colonies were both viable strategies for inducing and isolating transformants with full selection marker and inducible Cre recombinase cassette removal. There is a tradeoff depending on which strategy is employed between colony success percentage and time spent on the induction and isolation process, where the fastest method of direct plate induction yields a low percentage of successful isolates, and the slower method of liquid induction over multiple days and then plating yields a higher percentage of successful isolates. For all tests of the CRoxP system covered in the next section, 3 days in liquid induction medium followed by plating to singles on induction plates was used and the percentage of successful colonies varied from 20 to 80%, indicating that the integration site may play some role in the proportion of colonies that were successful.

### 3.2 Utilization of the CRoxP system to generate marker- and heterologous gene-less depigmented transformants of *P. celeri*

**FIGURE 6:**
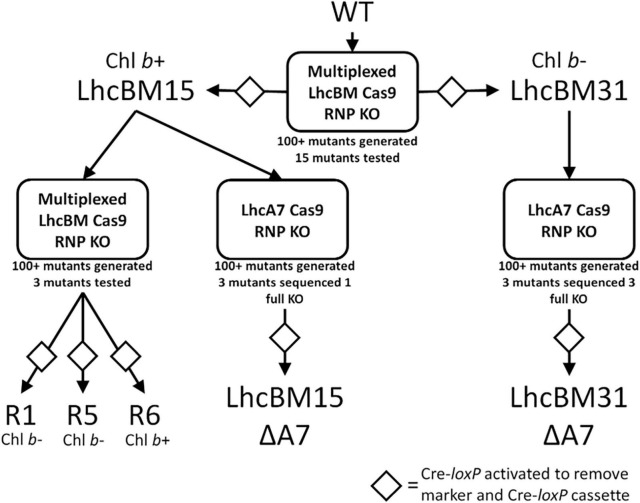
Flow diagram of transformant strains generated using the CRoxP system for gene knockouts followed by selection marker and Cre-recombinase cassette removal. The Cas9 guide target is listed in the boxes, below the boxes are the number of transformants generated for that transformation reaction, and of those transformants, how many were tested. The diamonds indicate activation of the inducible Cre-*loxP* system to remove the selection marker and Cre-recombinase cassette.

As discussed in the introduction, *P. celeri* has five different putative *LHCBM* genes and 9 different putative *LHCA* genes, the proteins of which constitute the LHCII and LHCI light harvesting antennae, respectively. Since *P. celeri* is diploid, this indicates that there are 28 distinct major LHC gene alleles that could be targeted for knockout by CRISPR Cas9 disruption to generate a depigmented strain by truncation of the LHCII and LHCI antennae. Little is currently known about the arrangement of the LHC antennae proteins in *P. celeri*, but light-harvesting cross section analyses and oxygen evolution experiments under high light suggest that it naturally truncates its LHC antennae based on light availability and intensity, and that the truncated versions remain efficient at very high light. Therefore, forcing *P. celeri* to remain in what mimics a truncated state by knocking out *LHCII* and *LHCI* genes could represent a path toward improving the performance of this strain in outdoor ponds. Since LHC antenna proteins naturally lend themselves to stacking mutations in a single strain to try to minimize pigment levels and balance photosystem light absorption, stacking LHC knockout in *P. celeri* represents a suitable test use case for the CRoxP system. Figure 6 presents a flow chart of key strains, where the CRoxP system was deployed, what the knockout targets were, how many colonies resulted from each transformation, and information about whether the strains possessed Chl *b* or not due to the knockout of LHCII genes.

#### 3.2.1 Multiplexed knockouts of *lhcBM* genes using CRoxP targeted to a region of shared homology

**FIGURE 7:**
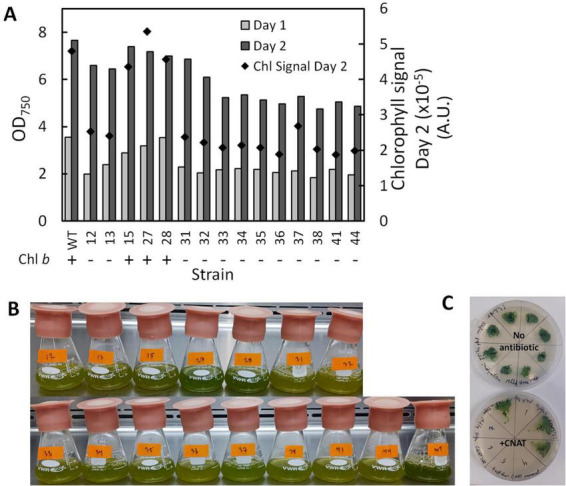
Culture density after one (light gray bars) and two (dark gray bars) days of growth using OD_750_ as a proxy (left *y*-axis) and mean chlorophyll signal in Arbitrary Units (A.U.) on a per cell basis (black diamonds, right *y*-axis) **(A)**, visual comparison of flask cultures after 2 days of growth showing the different pigmentation of the transformants vs. the wildtype (WT, bottom right-most flask) **(B)**, and example of results from double-plating colonies to assess selection marker and inducible Cre-recombinase cassette removal after induction of Cre recombinase **(C)**.

The LHCBMs in *P. celeri* exhibit high homology and share a considerable degree of sequence similarity. To target all LHCBMs simultaneously, a single guide was designed and employed in this study. Wild type (WT) *P. celeri* was first transformed using the CRoxP system where the Cas9 RNP was loaded with this multiplexed sgRNA, leading to a variety of transformants with different levels of depigmentation. The colonies generated by this transformation ranged in size and displayed different pigmentation levels. Because the goal was depigmentation, more pale green colonies were selected for analysis than dark green colonies. Figure 7A shows the results of a flask experiment on the selected strains from this transformation. Culture growth was analyzed using OD_750_ over 2 days (left *y*-axis) and pigmentation was analyzed on a single cell basis by flow cytometry (right *y*-axis). Of the analyzed colonies, 12 of the 15 lacked Chl *b* as determined by chlorophyll extraction and spectrophotometric quantification, as well as whole-cell absorption spectra analysis (see Supplementary Figure S1). The high occurrence of Chl *b*-less transformants was likely due to the colony selection process, and not necessarily representative of the population of transformants that resulted from the transformation. In flasks, the different pigmentation of the transformants was clearly visible compared to the WT (the lower right-most flask in the image, see Figure 7B). Based on culture growth and pigmentation, one Chl *b*- (strain LhcBM31) and one Chl *b*+ (strain LhcBM15) transformant were selected for further knockout stacking. Strain LhcBM15 was chosen as the Chl *b*+ candidate because it exhibited culture densities close to the WT with some level of depigmentation as seen in the mean chlorophyll signal per cell (Figure 7A, black diamonds, right *y*-axis) and visually by observing the flasks (Figure 7B). Strain LhcBM31 was chosen as the Chl *b*- candidate because it reached the highest culture densities among the Chl *b*- strains while also exhibiting mean chlorophyll signals per cell that were about 50% lower than those seen in the WT. In the strains selected for further knockout stacking, Cre was induced and successful removal of the selection marker/inducible Cre recombinase cassette was validated in clonal isolates by PCR and by double streaking on plates with and without antibiotics. Figure 7C shows an example plate utilizing the double streak strategy where the patches that did not grow on the antibiotic plates indicated success of Cre-*loxP*.

#### 3.2.2 Stacked KO transformants generated by the CRoxP system targeting *lhcBM* and *lhcA* genes in two *lhcBM* transformant strains

**FIGURE 8:**
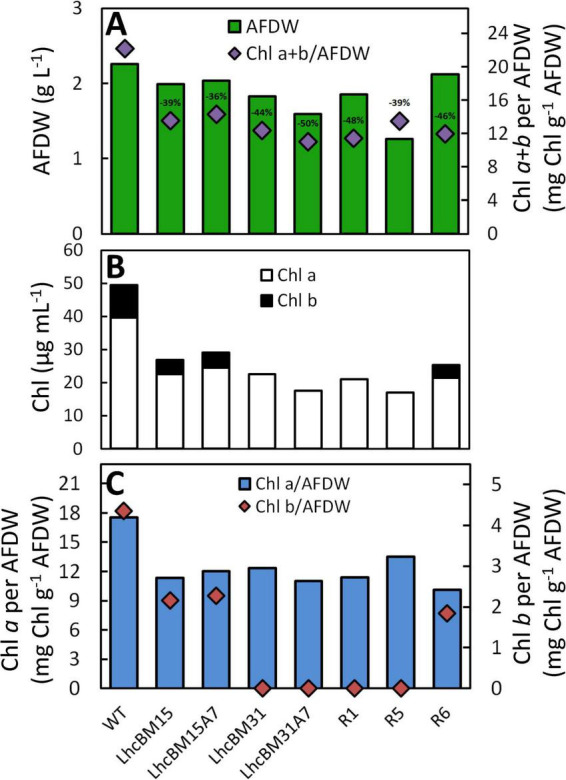
Results from flask experiments on wildtype (WT) and depigmented transformants of *P. celeri* after 2 days growth showing biomass concentration (g AFDW L^–1^) on the left *y*-axis (green bars), and chlorophylls *a* + *b* on a biomass basis (mg Chl g^–1^ AFDW) on the right *y*-axis (purple diamonds) **(A)**, chlorophyll concentrations per culture volume (μg Chl mL^–1^) **(B)**, and Chl *a* (blue bars) vs. Chl *b* (red diamonds) per biomass (mg Chl g^–1^ AFDW) on the left vs. right *y*-axis, respectively **(C)**. The percent reduction in Chl per biomass is included above the purple diamonds in A. Results are the averages of two separate flasks for each strain.

As shown in Figure 6, strain LhcBM15 was put through another round of multiplexed targeting of the *LHCBM* genes as in section 3.2.1 and separately through a transformation targeting the *LHCA7* gene only, both using the CRoxP system. Strain LhcBM31 was only put through another transformation targeting the *LHCA7* gene because the first round targeting the *LHCBM* genes had significantly reduced pigmentation and eliminated Chl *b* entirely. Table 1 summarizes the disruptions to the targeted genes from all transformations and Supplementary Table 1S provides information on the nature of each disruption and in which allele that disruption occurred. From the two transformations targeting the *LHCA7* gene in strains LhcBM15 and LhcBM31, two strains with successful knockouts of both alleles were chosen by validation using Sanger sequencing and designated LhcBM15A7 and LhcBM31A7. Due to the complex nature of the resulting transformants from the transformation targeting the 5 *LHCBM* genes simultaneously in the LhcBM15 strain, three transformants were selected by their depigmented appearance on patch plates (strains R1, R5, and R6). Figure 8 shows the results of a set of flask experiments performed in duplicate for the WT compared to the two parent strains described in section 3.2.1 (LhcBM15 and LhcBM31), and stacked transformants arising from those parent strains (LhcBM15A7, LhcBM31A7, R1, R5, and R6). Figure 8A shows a comparison of the culture densities in g AFDW L^–1^ after 2 days of growth (gray bars, left *y*-axis) as well as the Chl per AFDW (mg Chl *a* + *b* g^–1^ AFDW, black diamonds, right *y*-axis) measured spectrophotometrically. Compared to the WT, the transformants demonstrated 36–50% lower Chl on an AFDW basis. Two of the three strains tested from the second round of *LHCBM* multiplexed knockouts in strain LhcBM15 were Chl *b*-less, but strain R6 showed less chlorophyll on a biomass basis than the parent strain with higher biomass concentrations, which were similar to the WT. As shown in Table 1, the main difference between the parent strain LhcBM15 and R6 was the knock-out (KO) of the *LHCBM4* gene. When analyzing the growth using biomass concentration after 2 days as a proxy, it appeared Chl *b*-less transformants exhibited an impairment, at least when grown in small flask cultures. The different performance of the Chl *b*-less transformants suggests that not all Chl *b*-less strains were the same, which might have been expected if a disruption in an *LHCBM* gene was eliminating the LhcBM proteins from entering the chloroplast or thylakoid membrane and being populated by Chl *b*. Figure 8B compares Chl *a* and *b* concentrations of the cultures (μg Chl mL^–1^ culture) showing that the transformant cultures ranged from 40 to 60% of the chlorophyll concentrations on a culture volume basis compared to the WT. Figure 8C analyzes the Chl per biomass for Chl *a* (gray bars, left *y*-axis) and Chl *b* (black diamonds, right *y*-axis) independently to highlight that Chl *b*-lessness did not necessarily lead to a compensatory increase in Chl *a*.

**TABLE 1:** Summary of strains, genotypes, and Chl *b* status for the principal transformants of *P. celeri* in this study.

Strain	Major LHCII genes					LHC I gene	Chl *b*
	***lhcBM2***	***lhcBM3***	***lhcBM4***	***lhcBM6***	***lhcBM7***	***lhcA7***	
LhcBM15	WT/WT	WT/IF	X/WT	WT/WT	X/X	WT/WT	+
R1	WT/WT	X/IF	X/WT	X/IF	X/X	WT/WT	–
R5	WT/WT	X/IF	X/X	X/X	X/X	WT/WT	–
R6	WT/WT	?/IF	X/X	WT/WT	X/X	WT/WT	+
LhcBM15A7	WT/WT	WT/IF	X/WT	WT/WT	X/X	X/X	+
LhcBM31	WT/WT	IF/?	X/X	X/X	X/X	WT/WT	–
LhcBM31A7	WT/WT	IF/?	X/X	X/X	X/X	X/X	–

Each gene has two alleles with the status of each allele indicated as WT (wildtype), X (gene disruption resulting in either a stop codon or frame shift in amino acid translation), IF (In Frame mutation leading to insertion or deletion of amino acids at the cut site but normal translation after the mutation), or ? (PCR product missing either due to large DNA insertions or some unknown effect leading to absence of copies of that allele and therefore no sequencing data available by Sanger Sequencing). WT, wildtype; IF, In Frame mutation; X, gene knockout; ?, PCR product missing.

**FIGURE 9:**
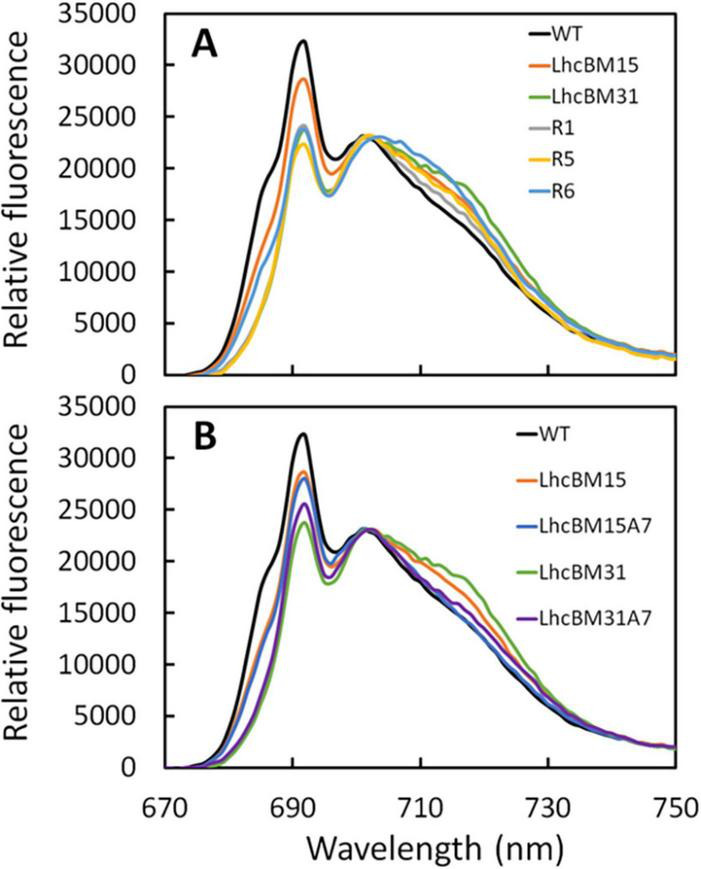
77K chlorophyll fluorescence chromatograms normalized to 702 nm of wildtype (WT) compared with LHCII multiplexed knockout transformants **(A)** and WT compared with LHCII multiplexed knockout transformants and their subsequent *lhcA7* knockout transformant daughter lines **(B)** of *P. celeri* grown in flask cultures after 2 days of growth. Results are the averages of two separate flasks for each strain. Chromatograms were normalized to 702 nm because it was found that normalization at the 692 peak or the 695 trough led to over emphasis on either the LHCI/PSI (∼705–730 nm) or the LHCII/PSII (∼675–700 nm) region’s differences, respectively.

As a relatively simple method for analyzing how LHCI and LHCII gene knockouts affected the organization of the LHCs, 77K fluorescence was performed on samples from the duplicate flask experiments. Figure 9A shows a comparison of the transformants in which LHCII genes were targeted by multiplexed knockout with the WT and Figure 9B shows a comparison between the WT, transformants with LHCII gene knockouts, and their daughter lines with an LHCI gene knockout. The chromatograms were normalized to 702 nm because it was found that normalization at the 692 peak or the 695 trough led to over emphasis on either the LHCI/PSI (∼705–730 nm) or the LHCII/PSII (∼675–700 nm) region’s differences, respectively. In Figure 9A, several key observations can be made about the nature of LHCII disruptions. First, all strains bearing Chl *b* possessed a characteristic signal between 675 and 685 nm, which the Chl *b*-less transformants lacked. This characteristic was also seen by Krishnan et al. (2023) with the deletion of cpSRP43, though that transformant did have some Chl *b*, albeit at a very reduced level compared to WT. Second, the overall peak area associated with the PSII/LHCII region between ∼675 and 700 nm was reduced in LHCII KO transformants, though the relative decrease was not necessarily consistent with the level of depigmentation per biomass. For instance, LhcBM15 and R5 both showed a 39% decrease in Chl per AFDW compared to WT, but the relative peak area of R5 in the PSII/LHCII region was dramatically different compared to LhcBM15. Third, elimination of LHCII proteins appeared to cause an increase in the relative peak area associated with PSI/LHCI between ∼705 and 730 nm. Among the LHCII KO transformants, the greatest increase in this region was in strain R6, which interestingly also had nearly the same biomass concentration of the WT as shown in Figure 8A. In Figure 9B, elimination of one of the *LHCI* genes (*LHCA7*) led to the expected outcome of decreasing the relative peak area associated with the PSI/LHCI region between ∼705 and 730 nm.

#### 3.2.3 Analysis of depigmented strains of *P. celeri* generated by the CRoxP system under diel light simulating a solar day in Mesa Arizona in June in ALGiSIM photobioreactors

**FIGURE 10:**
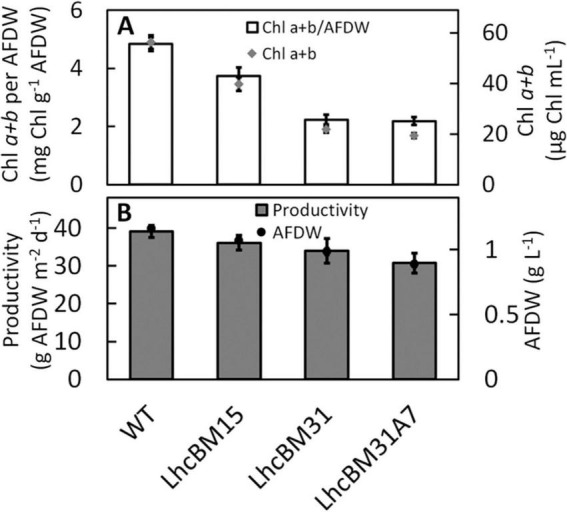
Comparisons of wildtype (WT), two LHCII multiplexed knockout transformants (LhcBM15 and LhcBM31), and an LHCII transformant with *lhcA7* also knocked out (LhcBM31A7) grown in the ALGiSIM photobioreactor under diel light simulating a solar day in Mesa Arizona in June. Chlorophylls a + b on a biomass basis (mg Chl g^–1^ AFDW, white bars) are on the left *y*-axis and chlorophyll per culture volume (μg mL^–1^, gray diamonds) is on the right *y*-axis **(A)**, areal productivity (g AFDW m^–2^ d^–1^, dark gray bars) is on the left *y*-axis and biomass concentration (g AFDW L^–1^, black circles) is on the right *y*-axis **(B)**. Error bars represent ± 1 standard deviation of three separate days of measurements on the same cultures.

Several transformants from sections 3.2.1 and 3.2.2 were selected for analysis under more pond relevant conditions simulating diel solar days in Mesa Arizona in May and diluting the cultures once per day (50% 1 h before sunset, see section 2.12 for additional culturing conditions for these experiments). The goal of this was to compare the biomass productivities of the transformants and their pigment levels as might be seen in an outdoor pond. Figure 10A compares the Chl on a biomass basis (mg Chl *a* + *b* g^–1^ AFDW, white bars, left *y*-axis) and a culture volume basis (μg Chl *a* + *b* mL^–1^, gray diamonds, right *y*-axis) for the WT, the two LHCII multiplex KO transformants (LhcBM15 and LhcBM31), and the LHCI/LHCII stacked KO transformant (LhcBM31A7). Figure 10B shows the areal biomass productivities (g AFDW m^–2^ d^–1^, gray bars, left *y*-axis), and biomass concentrations (g AFDW L^–1^, black circles, right *y*-axis). While the transformants did not reach the same areal biomass productivities seen in the WT, they still achieved very high productivities despite massively reduced Chl levels. For example, LhcBM31 had 53% less chlorophyll per biomass compared to WT but still achieved 34 g AFDW L^–1^ d^–1^ (13% lower than WT). Because the cultures were constantly mixed by magnetic stir bars and rapid bubbling, testing the relative performance of depigmented transformants in this type of laboratory setting may not accurately reflect the conditions in a pond. In most types of raceway ponds the cells spend periods either at the surface where they experience full sunlight, or deep in the pond where they experience significant shading, which is the condition in which depigmented strains are hypothesized to outperform WT ponds.

## 4 Discussion

### 4.1 CRoxP (Cas9 RNPs coupled with inducible Cre-loxP) system

**FIGURE 11:**
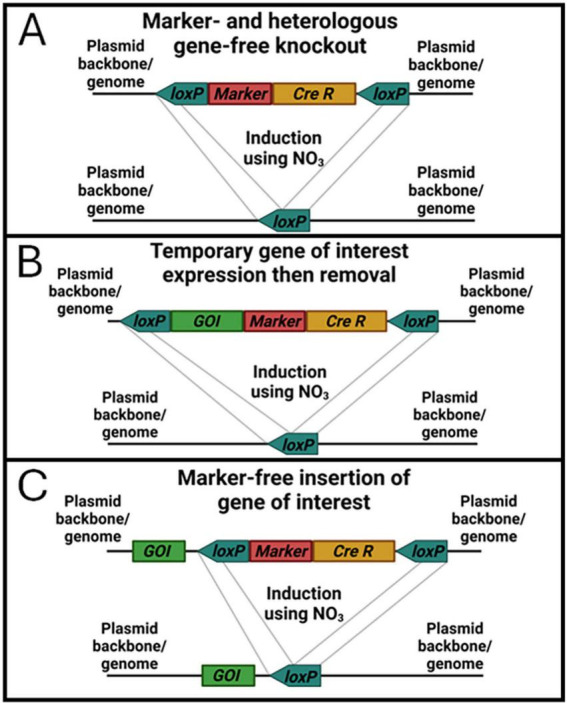
Schematic diagrams of three selection marker and inducible Cre recombinase cassettes with *loxP* sites oriented for different outcomes after induction of Cre recombinase. Box **(A)** depicts the simple linearized construct from this study that contains a selection marker and inducible Cre recombinase cassette between two *loxP* sites oriented in the same direction that result in removal of the selection marker and inducible Cre recombinase after activation of Cre recombinase. Box **(B)** shows a construct that also has a gene of interest (GOI) added between the *loxP* sites, which would be suitable for expressing a GOI in a transformant followed by removal of the gene along with the selection marker and Cre recombinase by Cre recombinase induction. Box **(C)** also shows a construct with a GOI included, but here the GOI is located outside the *loxP* sites. This construct would be suitable for expression of heterologous genes for producing novel products or phenotypes as well as to overexpress native genes by combining a native gene with a higher transcript promoter/terminator combination from the native genome.

As new strains of high-performance algae are discovered, engineering those strains to improve their characteristics is necessary to make novel products or increase their economic viability. Crucially, this engineering should be done in a manner that results in deployable transformants. Increasing the speed and efficiency at which strains can be transformed, tested, and transformed again is also key to generating next generation strains with stacked phenotypes. The novel CRoxP (Cas9 RNPs coupled with inducible CRe-loxP) system presented in this study provides a method for precise gene editing, selection marker recycling, and generation of selection marker- and heterologous gene-free transformants. By utilizing Cas9 RNPs for genome editing instead of a Cas9 expression construct, the need to tune Cas9 expression is eliminated, Cas9 toxicity and off-target editing are presumably reduced due to the transient nature of Cas9 RNPs present in the cell, and building the Cas9-sgRNA RNP *in vitro* allows *in vitro* validation of cutting efficiency with the same RNP used in actual transformations, thereby reducing the risk of problems with RNP formation *in vivo* from expression constructs. Combining Cas9 RNPs with an inducible Cre-*loxP* system built into the selection marker construct allows selection marker recycling and/or elimination after successful genome editing while simultaneously removing the inducible Cre recombinase cassette. Recycling the selection marker for multiple rounds of gene editing eliminates the need for multiple selection markers for the same organism and simplifies the research and development necessary to optimize selection markers for transformation in new strains. Eliminating the selection marker, inducible Cre recombinase cassette, and any other DNA not desired in the final strain by flanking it with *loxP* sites allows the generation of strains lacking any non-native genes, allowing outdoor deployment based on current regulations. Generating strains using CRoxP from the outset will significantly reduce the risk and time associated with recreating transformant strains generated by conventional methods. Figure 11 shows schematic diagrams of three different ways in which the CRoxP system can be deployed depending on the desired outcome within a strain. By positioning *loxP* sites in the same direction outside the selection marker and inducible Cre recombinase cassette (Figure 11A), activation of Cre recombinase yields a strain with only the plasmid backbone flanking a single *loxP* site. Placing a gene of interest inside (Figure 11B) or outside (Figure 11C) the *loxP* sites can also be useful depending on the research and/or strain development goals. By placing the *loxP* sites outside of a gene of interest cassette (Figure 11B), the function of that gene can be tested or utilized and then removed along with the selection marker and inducible Cre cassette. This configuration might be useful for cases where Cas9 expression is desired instead of using an RNP constructed *in vitro*, or where expression of a toxic enzyme is required on a temporary basis. Placing the *loxP* sites flanking the selection marker with a gene of interest outside (Figure 11C) will yield a strain without the selection marker or inducible Cre recombinase cassette but leaving behind the gene of interest. There are many cases in which this would be a desirable outcome such as overexpression of a native gene or introduction of novel enzymatic pathways.

**FIGURE 12:**
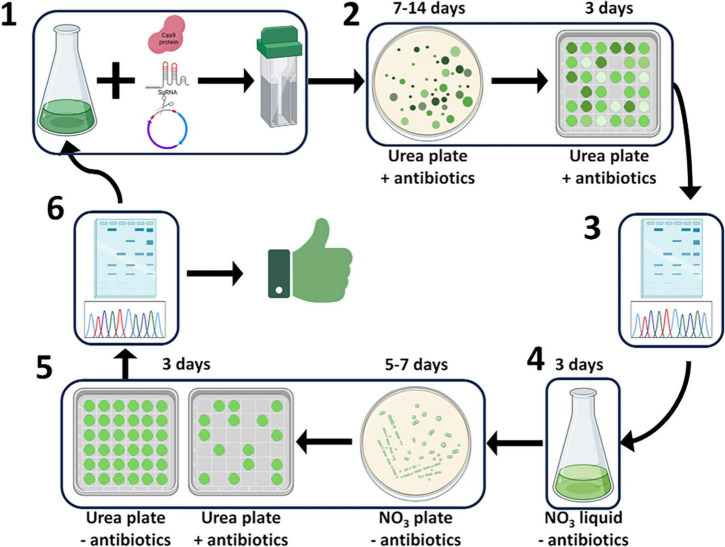
Schematic diagram of the CRoxP system deployed in *P. celeri* showing the steps and time per step to yield a selection marker- and inducible Cre recombinase-free transformant in 21–30 days. Step 1 depicts the transformation process where Cas9 RNPs and linearized selection marker + inducible Cre recombinase construct are electroporated into *P. celeri* cells. Step 2 depicts the transformant selection process on antibiotic plates where transformant colonies arise from selection marker integration into the cut site generated by the Cas9 RNP. Steps 3 and 6 depict transformant and successful Cre recombinase activity validation by Sanger sequencing, respectively. Step 4 is where Cre recombinase is induced by switching the nitrogen source to nitrate in liquid culture, leading to the removal of the selection marker and the inducible Cre recombinase cassette. Step 5 shows the isolation of cells and initial validation of Cre recombinase activity following induction where clonal isolates are double streaked onto plates with and without antibiotics, those colonies that do not grow on the antibiotic plate give strong evidence of successful Cre recombinase activity. After step 6, the transformants are either ready for another round of transformation using the same selection marker or are readily deployable into outdoor ponds because they lack selection markers and the heterologous Cre recombinase gene.

In the high biomass productivity marine green alga *P. celeri*, deployment of the CRoxP system allowed the knockout of genes and elimination of the selection marker and inducible Cre-*loxP* cassette, yielding a transformant ready for another round of transformation in as little as 21 days. Figure 12 shows a schematic diagram of CRoxP deployment in *P. celeri*. Conventional Cas9 RNP transformations follow steps 1 through 3 whereas the CRoxP system requires 11–13 additional days for selection marker and inducible Cre recombinase cassette removal and validation. See the figure legend for a full description of the CRoxP process steps. Section 3.1 of this study showed the development of the CRoxP system in *P. celeri*, validating the nitrate reductase promoter and terminator as a suitable inducible cassette to regulate Cre recombinase expression (section 3.1.1), and analyzing methods for Cre recombinase activation and transformant isolation (section 3.1.2) finding a tradeoff between speed and success rate between liquid culture induction (slower, higher success rate) and direct plating to clonal isolates on induction plates (faster, lower success rate). Future research on CRoxP deployment in *P. celeri* and other strains would benefit from genome resequencing for several reasons. First, resequencing would enable determination of off target effects caused by the Cas9 RNP as well as Cre recombinase. Second, resequencing would validate that no non-native genes were inadvertently incorporated into the genome, which is important for outdoor deployment based on regulatory requirements and safety concerns. Finally, resequencing would enable analyses of the stability of genome edits over time, which is important for desired trait stability.

### 4.2 Deployment of CRoxP for generation of depigmented strains of *P. celeri*

Because there are two alleles each of nine *LHCA* and five *LHCBM* genes in *P. celeri*, generating depigmented transformants with stacked knockouts was a suitable use-case for deployment of the CRoxP system. In this study, multiple depigmented transformants were generated using a multiplexed knockout of *lhcBM* genes. A subset of those transformants were analyzed, and two were selected for further transformations following marker and inducible Cre recombinase cassette removal. LhcBM15 (Chl *b*+) and LhcBM31 (Chl *b*-) went through a second round of transformation eliminating the *LHCA7* gene yielding strains LhcBM15A7 and LhcBM31A7. LhcBM15 was also put through another round of multiplexed attack of the *lhcBM* genes leading to strains R1 (Chl *b*-), R5 (Chl *b*-), and R6 (Chl *b*+). Depigmented strains varied from 39 to 50% less Chl *a*+*b* on a biomass basis compared to WT in flask cultures and up to 54% less (LhcBM31A7) when tested under diel solar simulating light. Strain R6 exhibited similar biomass accumulation in flask culture as WT with 43% less Chl *a*+*b* on a biomass basis. Chl b-lessness led to dissimilar phenotypes suggesting that not all Chl *b*-less strains were the same, but all appeared to have some level of growth impairment. 77K fluorescence of the cultures showed a wide variation in the PSII/LHCII region, and knocking out major LHCII antenna proteins (*lhcBM* genes) led to an increase in relative fluorescence of the PSI/LHCI region of the chromatogram. Knockout out the *LHCA7* gene caused the PSI/LHCI region to decrease back to a similar relative size as the WT. Combined, these results suggest that while many of the strains selected for testing in this study did not perform as well as the WT under the tested conditions, balancing the LHCI and LHCII antennae in depigmented transformants may be possible by generating stacked knockouts in a strain using the CRoxP system. This type of LHCII/LHCI balancing approach was shown by Krishnan et al. (2023) to slightly improve diel biomass productivity. Future research in this area should focus on scalable screening methods to quickly process large numbers of transformants to select superior candidates for further knockouts/photosystem balancing. Further physiological and biochemical analysis of these and other depigmented strains could also provide important information to optimize gene knockout combinations and thus, strain performance. Additionally, study on the structure of the PSI/LHCI and PSII/LHCII complexes in *P. celeri* would also help in designing knockout plans to systematically truncate and balance the photosystems.

## Data Availability

Publicly available datasets were analyzed in this study. These data can be found here: https://www.ncbi.nlm.nih.gov/, accession numbers AF330636 and PRJNA598876.
