# Supplementary material for: Leveraging CRISPR Cas9 RNPs and Cre-loxP in Picochlorum celeri for generation of field deployable strains and selection marker recycling
Source: Front Microbiol. 2025 Jul 14;16:1588625. doi: 10.3389/fmicb.2025.1588625 (PMC12301381; doi:10.3389/fmicb.2025.1588625)
Supplement: Supplementary file 1 [file Supplementary_file_1.docx]

Table S1. Details of gene disruptions by strain where P0 and P1 represent the two different diploid alleles in *P. celeri*. Blue strains in the left column were Chl b-less, alleles shaded orange were in-frame mutations, and red shading indicates genes that were disrupted by frame-shift mutations or selection marker insertions.


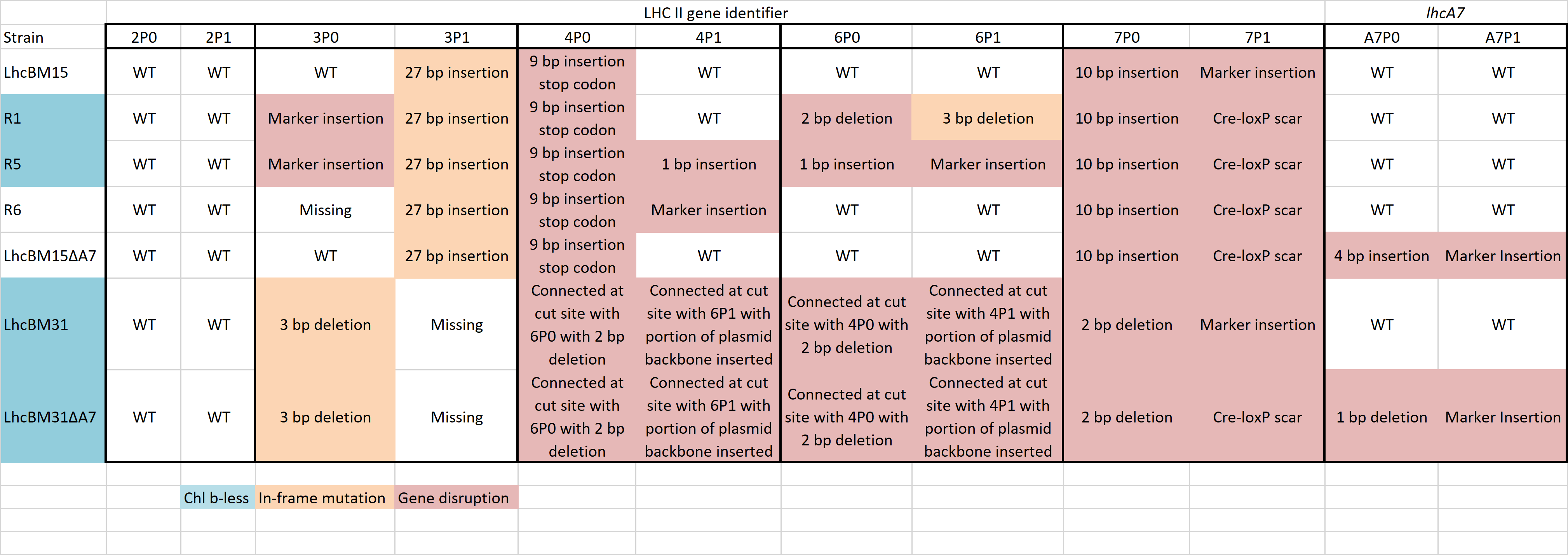


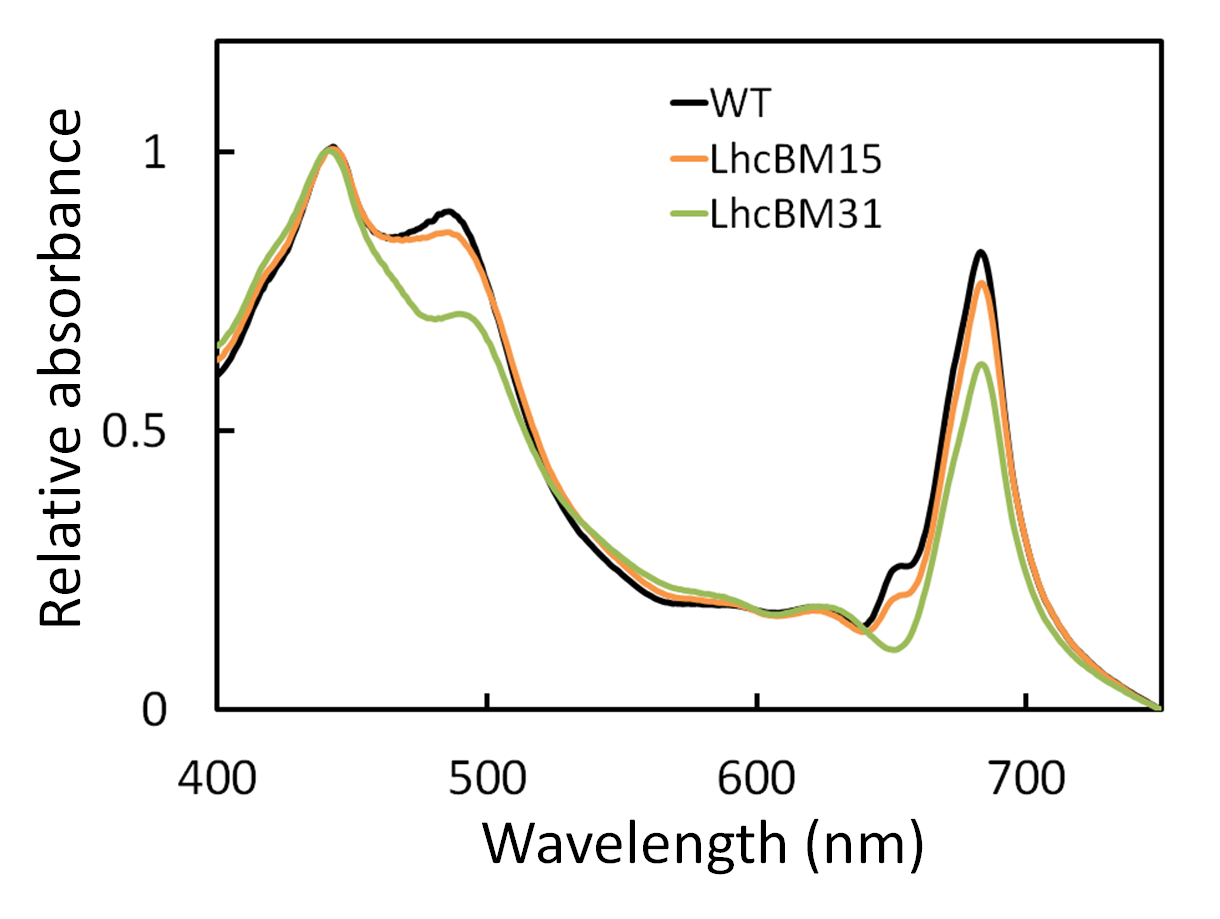


Figure S1. Whole-cell absorption spectra normalized to 440 nm for wildtype (WT) and two different LHCII multiplexed knockout mutants of *P. celeri* grown in the ALGiSIM photobioreactor. Strain LhcBM31 was Chl b-less and lacked the characteristic bump of Chl b at ~650nm seen in the Chl b-bearing WT and LhcBM15 strains.

pNRCreCNAT plasmid map:


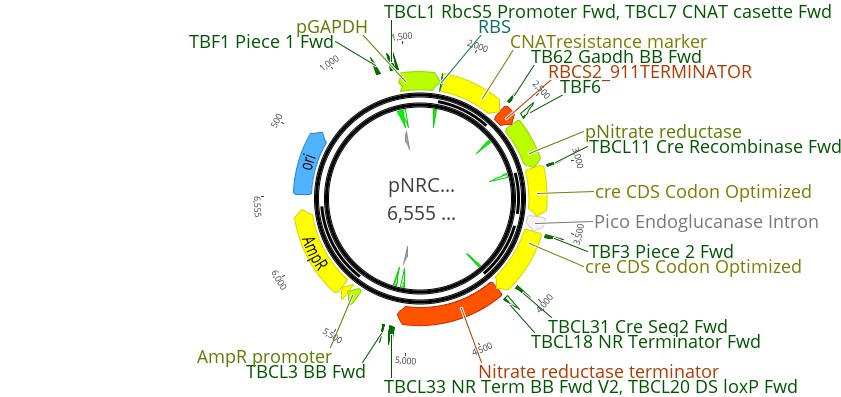


pNRCreCNAT full sequence:

AAAGGATCTTCTTGAGATCCTTTTTTTCTGCGCGTAATCTGCTGCTTGCAAACAAAAAAACCACCGCTACCAGCGGTGGTTTGTTTGCCGGATCAAGAGCTACCAACTCTTTTTCCGAAGGTAACTGGCTTCAGCAGAGCGCAGATACCAAATACTGTTCTTCTAGTGTAGCCGTAGTTAGCCCACCACTTCAAGAACTCTGTAGCACCGCCTACATACCTCGCTCTGCTAATCCTGTTACCAGTGGCTGCTGCCAGTGGCGATAAGTCGTGTCTTACCGGGTTGGACTCAAGACGATAGTTACCGGATAAGGCGCAGCGGTCGGGCTGAACGGGGGGTTCGTGCACACAGCCCAGCTTGGAGCGAACGACCTACACCGAACTGAGATACCTACAGCGTGAGCTATGAGAAAGCGCCACGCTTCCCGAAGGGAGAAAGGCGGACAGGTATCCGGTAAGCGGCAGGGTCGGAACAGGAGAGCGCACGAGGGAGCTTCCAGGGGGAAACGCCTGGTATCTTTATAGTCCTGTCGGGTTTCGCCACCTCTGACTTGAGCGTCGATTTTTGTGATGCTCGTCAGGGGGGCGGAGCCTATGGAAAAACGCCAGCAACGCGGCCTTTTTACGGTTCCTGGCCTTTTGCTGGCCTTTTGCTCACATGTTCTTTCCTGCGTTATCCCCTGATTCTGTGGATAACCGTATTACCGCCTTTGAGTGAGCTGATACCGCTCGCCGCAGCCGAACGACCGAGCGCAGCGAGTCAGTGAGCGAGGAAGCGGAAGGCGAGAGTAGGGAACTGCCAGGCATCAAACTAAGCAGAAGGCCCCTGACGGATGGCCTTTTTGCGTTTCTACAAACTCTTTCTGTGTTGTAAAACGACGGCCAGTCTTAAGCTCGGGCCCCCTGGGCGGTTCTGATAACGAGTAATCGTTAATCCGCAAATAACGTAAAAACCCGCTTCGGCGGGTTTTTTTATGGGGGGAGTTTAGGGAAAGAGCATTTGTCAGAATATTTAAGGGCGCCTGTCACTTTGCTTGATATATGAGAATTATTTAACCTTATAAATGAGAAAAAAGCAACGCACTTTAAATAAGATACGTTGCTTTTTCGATTGATGAACACCTATAATTAAACTATTCATCTATTATTTATGATTTTTTGTATATACAATATTTCTAGTTTGTTAAAGAGAATTAAGAAAATAAATCTCGAAAATAATAAAGGGAAAATCAGTTTTTGATATCAAAATTATACATGTCAACGATAATACAAAATATAATACAAACTATAAGATGTTATCAGTATTTATTATGCATTTAGAATAAATTTTGTGTCGCCCTTCGCTGAAGGCGCGCCGAATTCGCTAGCATAACTTCGTATAGCATACATTATACGAAGTTATATCGATGTATGTGATATAATATACCTGCCGTCAACGAGTCACGTTCAAATGTACACACGCCTAGAGTGGTGCGGCAATTCAATCTATGTGCGAGAATATGTCGCTTTGTTTCCTAATAGATTAGATGATGTGCATGCTAGGTGGAGAGTATCGACCTCCACATGACTGCAGGAAAGCATTCCTTTTCGAAGTAGTCCCATCAATGACTCGGCTGGAAAGGATGAAGGTGCCTTGTCCATCCCTCCCCATCAAAGGCACCGTGTGATGGACAGATAACAAATAGAGCCATGTTGTATCGAAATAGTTCGTCCCATCTTATCGGATAAGGGAGTTCCTTCATCAAACTCCTGGAGCATTATCAAATTCCGCTTACTTTGCTACCTCAATCAAGTCTATTTAAATTTCAAAATGGCTTCTACAGGAACATTGGATGATACAGCTTACAGATACAGAACATCTGTGCCAGGAGATGCTGAGGCTATTGAGGCTTTGGATGGATCTTTCACAACAGATACAGTGTTCAGAGTGACAGCTACAGGAGATGGATTCACATTGAGAGAGGTGCCAGTGGATCCACCATTGACAAAGGTGTTCCCAGATGATGAGTCTGATGATGAGTCTGATGCTGGAGAGGATGGAGATCCAGATTCTAGAACATTCGTGGCTTACGGAGATGATGGAGATTTGGCTGGATTCGTGGTGGTGTCTTACTCTGGATGGAACAGAAGATTGACAGTGGAGGATATTGAGGTGGCTCCAGAGCATAGAGGACATGGAGTGGGAAGAGCTTTGATGGGATTGGCTACAGAGTTCGCTAGAGAGAGAGGAGCTGGACATTTGTGGTTGGAGGTGACAAACGTGAACGCTCCAGCTATTCATGCTTACAGAAGAATGGGATTCACATTGTGCGGATTGGATACAGCTTTGTACGATGGAACAGCTTCTGATGGAGAGCAGGCTTTGTACATGTCTATGCCATGCCCATAAGCGGCCGCGTGATCCAGGTATAAACAAGGGATGAAAAAATATCAAATCTAATCAAAATTTTACAATGTATATGATACTCTGACATGTACTCCATTCGATATGACAAATGGCATGTAAAATATGTATGATTGTTACAGTTGTTCTTATTCATAGTGTACATGGAGTGCCGCGGTTCCGAATTGTATATAAAGCCATGGTCATATGTTTGACCTCATGCATGGATGAACATCAATTTGCATGAAGATCAGCGATTGCCTATACAGTATCGATGGATGCGTTGTCATTTTCAAAAAGATACATCTTTAAGAATATCAGCAACCATATGGGCCTGCTCTTGTATCAAATGGATAAGAGGAAGCATGATCAAAGTCCAGACCTACTACTCCTACCCTGGCTCACGAGGAGATTCAGTTTGAGTCTGAGTCCACTGACTCACATACATCAGGGTTTTCCATGAACGATGAACAGATGGAAGGCGTTGCTGATAATCTAATTTCACATTTAATTACATTTCATGATTTTTAAAACACATCAGTTGTAGTCTTTTGGCCTCAATCTCACTGTGGGATTTTTCAACTGGGTGATACTGTCTTCAAGATGTCCAATCTTCTGACCGTACACCAAAATTTGCCTGCATTGCCGGTCGATGCAACGAGTGATGAGGTTCGCAAGAACCTGATGGACATGTTCAGGGATCGCCAGGCGTTTTCTGAGCATACCTGGAAAATGCTTCTGTCCGTTTGCCGGTCGTGGGCGGCATGGTGCAAGTTGAATAACCGGAAATGGTTTCCCGCAGAACCTGAAGATGTTCGCGATTATCTTCTCTATCTTCAGGCGCGCGGTCTGGCAGTAAAAACTATCCAGCAACATTTGGGCCAGCTGAACATGCTTCATCGTCGGTCCGGGCTGCCACGACCAAGTGACAGCAATGCTGTTTCACTGGTTATGCGGCGGATCCGAAAAGAAAACGTTGATGCCGGTGAACGTGCAAAACAGGCTCTCGCGTTCGAACGCACTGATTTCGACCAGGTGAGTTCAATGCGACAGTTGCATCTGTAGCTTCGTTATACATAACCAGTTGCCATACAACCATGTGCGAAGAAGCCCCCTGGTTGGCTACATGCCAAGAGTTTGAGAGAGTTTTGATGTATTTCACTTCTGCTGCAGGTTCGTTCACTCATGGAAAATAGCGATCGCTGCCAGGATATACGTAATCTGGCATTTCTGGGGATTGCTTATAACACCCTGCTTCGTATAGCCGAAATTGCCAGGATCAGGGTTAAAGATATCTCACGTACTGACGGTGGGAGAATGCTTATCCATATTGGCAGAACGAAAACGCTGGTTAGCACCGCAGGTGTAGAGAAGGCACTTAGCCTGGGGGTAACTAAACTGGTCGAGCGATGGATTTCCGTCTCTGGTGTAGCTGATGATCCGAATAACTACCTGTTTTGCCGGGTCAGAAAAAATGGTGTTGCCGCGCCATCTGCCACCAGCCAGCTTTCAACTCGCGCCCTGGAAGGGATTTTTGAAGCAACTCATCGATTGATTTACGGCGCTAAGGATGACTCTGGTCAGAGATACCTGGCCTGGTCTGGACACAGTGCCCGTGTCGGAGCCGCGCGAGATATGGCCCGCGCTGGAGTTTCAATACCGGAGATCATGCAAGCTGGTGGCTGGACCAATGTAAATATTGTCATGAACTATATCCGTAACCTGGATAGTGAAACAGGGGCAATGGTGCGCCTGCTGGAAGATGGCGATTAGGTTCTTGCATGGCCTTTTGATTGCTGATTTAATGAAAGTTTACATTCCATTTAATAATTCATAGCGCCCGTGTGTGTCAAATAAAATCAAAGCATTACAAAATCTCTACATTCAAAACCGTTTGAATGGCCTTGTCTCATGAAAACATAATCCAATCAAAGCGAAGAATAACCAGGCAGTAAACTTATACTCCTTGCTTGAATCTTCCTTTTGGTATGTCACAATGGAACCAATGCATGCAACCAACCCCATCAAGAATCCAACACAATACCTAAACTGTAGCCAAGGAGTTGTCAGCTCCTGCCAAACTGTACGCTTATTCTTATTACTCTGTACTGACGGTTCTTTCGGTTGTTGCTCTACACCCTTCCTGCTTTTCCGCCGTCTCAGCTCTACAGCAGCACCAGTCTTGGCAGAGGACCTAGCTTCAGGCATACTCAATACGTTTTCCACAGAAATCTCCGTTTCTTCGAGAATGGGCGTAAATATCGTCAATATTAAATTCGAACAAAAATTAACGCTGCAAGTACAGTGAAATTCAAAATATAGATTCAATCTGCCCAAACCAAACCTAGGGCACGGTCATATGACGCAACCCAGACTCAATTGCAATTCTGAGTCTGCAGACTCCTCATTCATTCATCATGGATCCGATTGAGCTTTGACACCCATGATGGTGGATGGACGATATCTCATACTCGCTCTTATTCTCATGTATGTATCGCATCGGATGAATTGACTGTGCCAGCCTGGAGAAGAAGAAGTCTTGGACCAATGCATTATTACTGCACCATTACCAGGCCTTGGAAAGATCCACAGAGACTTGTGCATATGATTGATTACAACTCTAGCCTTCTAGTGTACATTATATTTTAACATCGAAACGCAAATTTACATGAAAAAAAATCGTTATTGTTACTATCAAATCATCAAACTTCCATGTCAGATTCCTACATGGAGTGCGAAGATCATAACTTCGTATAGCATACATTATACGAAGTTATAGATCTTCGACCTCGAGCGGGGTACCCGACTAGTCCTGCAGGGGCGCGCCCGTCAAAAGGGCGACACCCCATAATTAGCCCGGGCGAAAGGCCCAGTCTTTCGACTGAGCCTTTCGTTTTATTTGATGCCTGGCAGTTCCCTACTCTCGCATGGGGAGTCCCCACACTACCATCGGCGCTACGGCGTTTCACTTCTGAGTTCGGCATGGGGTCAGGTGGGACCACCGCGCTACTGCCGCCAGGCAAACAAGGGGTGTTATGAGCCATATTCAGGTATAAATGGGCTCGCGATAATGTTCAGAATTGGTTAATTGGTTGTAACACTGACCCCTATTTGTTTATTTTTCTAAATACATTCAAATATGTATCCGCTCATGAGACAATAACCCTGATAAATGCTTCAATAATATTGAAAAAGGAAGAATATGAGTATTCAACATTTCCGTGTCGCCCTTATTCCCTTTTTTGCGGCATTTTGCCTTCCTGTTTTTGCTCACCCAGAAACGCTGGTGAAAGTAAAAGATGCTGAAGATCAGTTGGGTGCACGAGTGGGTTACATCGAACTGGATCTCAACAGCGGTAAGATCCTTGAGAGTTTTCGCCCCGAAGAACGTTTTCCAATGATGAGCACTTTTAAAGTTCTGCTATGTGGCGCGGTATTATCCCGTATTGACGCCGGGCAAGAGCAACTCGGTCGCCGCATACACTATTCTCAGAATGACTTGGTTGAGTACTCACCAGTCACAGAAAAGCATCTTACGGATGGCATGACAGTAAGAGAATTATGCAGTGCTGCCATAACCATGAGTGATAACACTGCGGCCAACTTACTTCTGACAACGATCGGAGGACCGAAGGAGCTAACCGCTTTTTTGCACAACATGGGGGATCATGTAACTCGCCTTGATCGTTGGGAACCGGAGCTGAATGAAGCCATACCAAACGACGAGCGTGACACCACGATGCCTGTAGCGATGGCAACAACGTTGCGCAAACTATTAACTGGCGAACTACTTACTCTAGCTTCCCGGCAACAATTAATAGACTGGATGGAGGCGGATAAAGTTGCAGGACCACTTCTGCGCTCGGCCCTTCCGGCTGGCTGGTTTATTGCTGATAAATCCGGAGCCGGTGAGCGTGGTTCTCGCGGTATCATCGCAGCGCTGGGGCCAGATGGTAAGCCCTCCCGTATCGTAGTTATCTACACGACGGGGAGTCAGGCAACTATGGATGAACGAAATAGACAGATCGCTGAGATAGGTGCCTCACTGATTAAGCATTGGTAAGCAGAGCATTACGCTGACTTGACGGGACGGCGCAAGCTCATGACCAAAATCCCTTAACGTGAGTTACGCGCGCGTCGTTCCACTGAGCGTCAGACCCCGTAGAAAAGATC

pNRCreBle plasmid map:


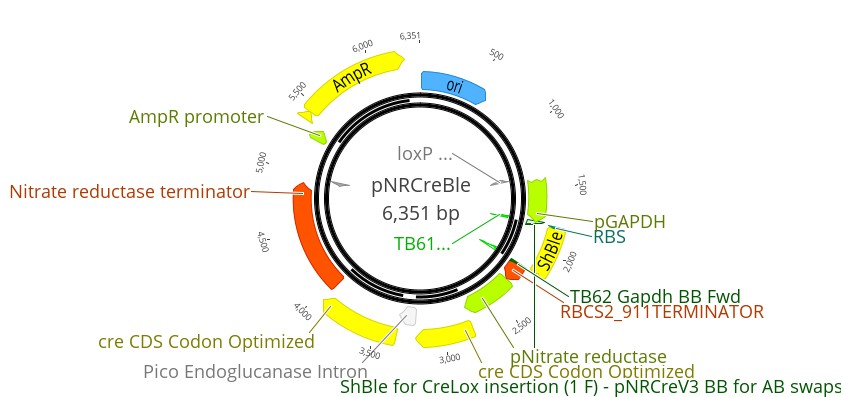


pNRCreBle full sequence:

AAAGGATCTTCTTGAGATCCTTTTTTTCTGCGCGTAATCTGCTGCTTGCAAACAAAAAAACCACCGCTACCAGCGGTGGTTTGTTTGCCGGATCAAGAGCTACCAACTCTTTTTCCGAAGGTAACTGGCTTCAGCAGAGCGCAGATACCAAATACTGTTCTTCTAGTGTAGCCGTAGTTAGCCCACCACTTCAAGAACTCTGTAGCACCGCCTACATACCTCGCTCTGCTAATCCTGTTACCAGTGGCTGCTGCCAGTGGCGATAAGTCGTGTCTTACCGGGTTGGACTCAAGACGATAGTTACCGGATAAGGCGCAGCGGTCGGGCTGAACGGGGGGTTCGTGCACACAGCCCAGCTTGGAGCGAACGACCTACACCGAACTGAGATACCTACAGCGTGAGCTATGAGAAAGCGCCACGCTTCCCGAAGGGAGAAAGGCGGACAGGTATCCGGTAAGCGGCAGGGTCGGAACAGGAGAGCGCACGAGGGAGCTTCCAGGGGGAAACGCCTGGTATCTTTATAGTCCTGTCGGGTTTCGCCACCTCTGACTTGAGCGTCGATTTTTGTGATGCTCGTCAGGGGGGCGGAGCCTATGGAAAAACGCCAGCAACGCGGCCTTTTTACGGTTCCTGGCCTTTTGCTGGCCTTTTGCTCACATGTTCTTTCCTGCGTTATCCCCTGATTCTGTGGATAACCGTATTACCGCCTTTGAGTGAGCTGATACCGCTCGCCGCAGCCGAACGACCGAGCGCAGCGAGTCAGTGAGCGAGGAAGCGGAAGGCGAGAGTAGGGAACTGCCAGGCATCAAACTAAGCAGAAGGCCCCTGACGGATGGCCTTTTTGCGTTTCTACAAACTCTTTCTGTGTTGTAAAACGACGGCCAGTCTTAAGCTCGGGCCCCCTGGGCGGTTCTGATAACGAGTAATCGTTAATCCGCAAATAACGTAAAAACCCGCTTCGGCGGGTTTTTTTATGGGGGGAGTTTAGGGAAAGAGCATTTGTCAGAATATTTAAGGGCGCCTGTCACTTTGCTTGATATATGAGAATTATTTAACCTTATAAATGAGAAAAAAGCAACGCACTTTAAATAAGATACGTTGCTTTTTCGATTGATGAACACCTATAATTAAACTATTCATCTATTATTTATGATTTTTTGTATATACAATATTTCTAGTTTGTTAAAGAGAATTAAGAAAATAAATCTCGAAAATAATAAAGGGAAAATCAGTTTTTGATATCAAAATTATACATGTCAACGATAATACAAAATATAATACAAACTATAAGATGTTATCAGTATTTATTATGCATTTAGAATAAATTTTGTGTCGCCCTTCGCTGAAGGCGCGCCGAATTCGCTAGCATAACTTCGTATAGCATACATTATACGAAGTTATATCGATGTATGTGATATAATATACCTGCCGTCAACGAGTCACGTTCAAATGTACACACGCCTAGAGTGGTGCGGCAATTCAATCTATGTGCGAGAATATGTCGCTTTGTTTCCTAATAGATTAGATGATGTGCATGCTAGGTGGAGAGTATCGACCTCCACATGACTGCAGGAAAGCATTCCTTTTCGAAGTAGTCCCATCAATGACTCGGCTGGAAAGGATGAAGGTGCCTTGTCCATCCCTCCCCATCAAAGGCACCGTGTGATGGACAGATAACAAATAGAGCCATGTTGTATCGAAATAGTTCGTCCCATCTTATCGGATAAGGGAGTTCCTTCATCAAACTCCTGGAGCATTATCAAATTCCGCTTACTTTGCTACCTCAATCAAGTCTATTTAAATTTCAAAATGGCTAAGTTGACTTCTGCTGTGCCAGTGTTGACTGCTAGAGATGTGGCTGGAGCTGTGGAGTTCTGGACTGATAGATTGGGATTCTCTCGTGATTTCGTGGAGGATGATTTCGCTGGAGTGGTGAGAGATGATGTGACTTTGTTCATCTCTGCTGTGCAGGATCAGGTGGTGCCAGATAACACTTTGGCTTGGGTGTGGGTGAGAGGATTGGATGAGTTGTACGCTGAGTGGTCTGAGGTGGTTTCTACTAACTTCAGAGATGCTTCTGGACCAGCTATGACTGAGATCGGAGAGCAGCCCTGGGGAAGAGAGTTCGCTTTGAGAGATCCAGCCGGAAACTGCGTGCACTTCGTGGCTGAGGAGCAGGATTAAGCGGCCGCGTGATCCAGGTATAAACAAGGGATGAAAAAATATCAAATCTAATCAAAATTTTACAATGTATATGATACTCTGACATGTACTCCATTCGATATGACAAATGGCATGTAAAATATGTATGATTGTTACAGTTGTTCTTATTCATAGTGTACATGGAGTGCCGCGGTTCCGAATTGTATATAAAGCCATGGTCATATGTTTGACCTCATGCATGGATGAACATCAATTTGCATGAAGATCAGCGATTGCCTATACAGTATCGATGGATGCGTTGTCATTTTCAAAAAGATACATCTTTAAGAATATCAGCAACCATATGGGCCTGCTCTTGTATCAAATGGATAAGAGGAAGCATGATCAAAGTCCAGACCTACTACTCCTACCCTGGCTCACGAGGAGATTCAGTTTGAGTCTGAGTCCACTGACTCACATACATCAGGGTTTTCCATGAACGATGAACAGATGGAAGGCGTTGCTGATAATCTAATTTCACATTTAATTACATTTCATGATTTTTAAAACACATCAGTTGTAGTCTTTTGGCCTCAATCTCACTGTGGGATTTTTCAACTGGGTGATACTGTCTTCAAGATGTCCAATCTTCTGACCGTACACCAAAATTTGCCTGCATTGCCGGTCGATGCAACGAGTGATGAGGTTCGCAAGAACCTGATGGACATGTTCAGGGATCGCCAGGCGTTTTCTGAGCATACCTGGAAAATGCTTCTGTCCGTTTGCCGGTCGTGGGCGGCATGGTGCAAGTTGAATAACCGGAAATGGTTTCCCGCAGAACCTGAAGATGTTCGCGATTATCTTCTCTATCTTCAGGCGCGCGGTCTGGCAGTAAAAACTATCCAGCAACATTTGGGCCAGCTGAACATGCTTCATCGTCGGTCCGGGCTGCCACGACCAAGTGACAGCAATGCTGTTTCACTGGTTATGCGGCGGATCCGAAAAGAAAACGTTGATGCCGGTGAACGTGCAAAACAGGCTCTCGCGTTCGAACGCACTGATTTCGACCAGGTGAGTTCAATGCGACAGTTGCATCTGTAGCTTCGTTATACATAACCAGTTGCCATACAACCATGTGCGAAGAAGCCCCCTGGTTGGCTACATGCCAAGAGTTTGAGAGAGTTTTGATGTATTTCACTTCTGCTGCAGGTTCGTTCACTCATGGAAAATAGCGATCGCTGCCAGGATATACGTAATCTGGCATTTCTGGGGATTGCTTATAACACCCTGCTTCGTATAGCCGAAATTGCCAGGATCAGGGTTAAAGATATCTCACGTACTGACGGTGGGAGAATGCTTATCCATATTGGCAGAACGAAAACGCTGGTTAGCACCGCAGGTGTAGAGAAGGCACTTAGCCTGGGGGTAACTAAACTGGTCGAGCGATGGATTTCCGTCTCTGGTGTAGCTGATGATCCGAATAACTACCTGTTTTGCCGGGTCAGAAAAAATGGTGTTGCCGCGCCATCTGCCACCAGCCAGCTTTCAACTCGCGCCCTGGAAGGGATTTTTGAAGCAACTCATCGATTGATTTACGGCGCTAAGGATGACTCTGGTCAGAGATACCTGGCCTGGTCTGGACACAGTGCCCGTGTCGGAGCCGCGCGAGATATGGCCCGCGCTGGAGTTTCAATACCGGAGATCATGCAAGCTGGTGGCTGGACCAATGTAAATATTGTCATGAACTATATCCGTAACCTGGATAGTGAAACAGGGGCAATGGTGCGCCTGCTGGAAGATGGCGATTAGGTTCTTGCATGGCCTTTTGATTGCTGATTTAATGAAAGTTTACATTCCATTTAATAATTCATAGCGCCCGTGTGTGTCAAATAAAATCAAAGCATTACAAAATCTCTACATTCAAAACCGTTTGAATGGCCTTGTCTCATGAAAACATAATCCAATCAAAGCGAAGAATAACCAGGCAGTAAACTTATACTCCTTGCTTGAATCTTCCTTTTGGTATGTCACAATGGAACCAATGCATGCAACCAACCCCATCAAGAATCCAACACAATACCTAAACTGTAGCCAAGGAGTTGTCAGCTCCTGCCAAACTGTACGCTTATTCTTATTACTCTGTACTGACGGTTCTTTCGGTTGTTGCTCTACACCCTTCCTGCTTTTCCGCCGTCTCAGCTCTACAGCAGCACCAGTCTTGGCAGAGGACCTAGCTTCAGGCATACTCAATACGTTTTCCACAGAAATCTCCGTTTCTTCGAGAATGGGCGTAAATATCGTCAATATTAAATTCGAACAAAAATTAACGCTGCAAGTACAGTGAAATTCAAAATATAGATTCAATCTGCCCAAACCAAACCTAGGGCACGGTCATATGACGCAACCCAGACTCAATTGCAATTCTGAGTCTGCAGACTCCTCATTCATTCATCATGGATCCGATTGAGCTTTGACACCCATGATGGTGGATGGACGATATCTCATACTCGCTCTTATTCTCATGTATGTATCGCATCGGATGAATTGACTGTGCCAGCCTGGAGAAGAAGAAGTCTTGGACCAATGCATTATTACTGCACCATTACCAGGCCTTGGAAAGATCCACAGAGACTTGTGCATATGATTGATTACAACTCTAGCCTTCTAGTGTACATTATATTTTAACATCGAAACGCAAATTTACATGAAAAAAAATCGTTATTGTTACTATCAAATCATCAAACTTCCATGTCAGATTCCTACATGGAGTGCGAAGATCATAACTTCGTATAGCATACATTATACGAAGTTATAGATCTTCGACCTCGAGCGGGGTACCCGACTAGTCCTGCAGGGGCGCGCCCGTCAAAAGGGCGACACCCCATAATTAGCCCGGGCGAAAGGCCCAGTCTTTCGACTGAGCCTTTCGTTTTATTTGATGCCTGGCAGTTCCCTACTCTCGCATGGGGAGTCCCCACACTACCATCGGCGCTACGGCGTTTCACTTCTGAGTTCGGCATGGGGTCAGGTGGGACCACCGCGCTACTGCCGCCAGGCAAACAAGGGGTGTTATGAGCCATATTCAGGTATAAATGGGCTCGCGATAATGTTCAGAATTGGTTAATTGGTTGTAACACTGACCCCTATTTGTTTATTTTTCTAAATACATTCAAATATGTATCCGCTCATGAGACAATAACCCTGATAAATGCTTCAATAATATTGAAAAAGGAAGAATATGAGTATTCAACATTTCCGTGTCGCCCTTATTCCCTTTTTTGCGGCATTTTGCCTTCCTGTTTTTGCTCACCCAGAAACGCTGGTGAAAGTAAAAGATGCTGAAGATCAGTTGGGTGCACGAGTGGGTTACATCGAACTGGATCTCAACAGCGGTAAGATCCTTGAGAGTTTTCGCCCCGAAGAACGTTTTCCAATGATGAGCACTTTTAAAGTTCTGCTATGTGGCGCGGTATTATCCCGTATTGACGCCGGGCAAGAGCAACTCGGTCGCCGCATACACTATTCTCAGAATGACTTGGTTGAGTACTCACCAGTCACAGAAAAGCATCTTACGGATGGCATGACAGTAAGAGAATTATGCAGTGCTGCCATAACCATGAGTGATAACACTGCGGCCAACTTACTTCTGACAACGATCGGAGGACCGAAGGAGCTAACCGCTTTTTTGCACAACATGGGGGATCATGTAACTCGCCTTGATCGTTGGGAACCGGAGCTGAATGAAGCCATACCAAACGACGAGCGTGACACCACGATGCCTGTAGCGATGGCAACAACGTTGCGCAAACTATTAACTGGCGAACTACTTACTCTAGCTTCCCGGCAACAATTAATAGACTGGATGGAGGCGGATAAAGTTGCAGGACCACTTCTGCGCTCGGCCCTTCCGGCTGGCTGGTTTATTGCTGATAAATCCGGAGCCGGTGAGCGTGGTTCTCGCGGTATCATCGCAGCGCTGGGGCCAGATGGTAAGCCCTCCCGTATCGTAGTTATCTACACGACGGGGAGTCAGGCAACTATGGATGAACGAAATAGACAGATCGCTGAGATAGGTGCCTCACTGATTAAGCATTGGTAAGCAGAGCATTACGCTGACTTGACGGGACGGCGCAAGCTCATGACCAAAATCCCTTAACGTGAGTTACGCGCGCGTCGTTCCACTGAGCGTCAGACCCCGTAGAAAAGATC

Primers used in this study:

| Name | Sequence (with extension) |
| --- | --- |
| TB61 Gapdh BB Rev | TTTGAAATTTAAATAGACTTGATTGAGGTAG |
| TB62 Gapdh BB Fwd | TAAGCGGCCGCGTGATC |
| TBCL1 RbcS5 Promoter Fwd | ACATTATACGAAGTTATATCGATGTATGTGATATAATATACC |
| TBCL3 BB Fwd | CGACTAGTCCTGCAGGGGC |
| TBCL4 BB loxP Rev | TACATCGATATAACTTCGTATAATGTATGCTATACGAAGTTATGCTAGCGAATTCGGCGC |
| TBCL7 CNAT casette Fwd | GCCGTCAACGAGTCACGTTC |
| TBCL8 CNAT Casette Rev | ACTCCATGTACACTATGAATAAGAAC |
| TBCL11 Cre Recombinase Fwd | ATGTCCAATCTTCTGACCG |
| TBCL12 Cre Recombinase Rev | CCCCTGCAGGACTAGTCGGGTACCCCGCTCGAGG |
| TBCL13 Cre recombinase only Rev | CTAATCGCCATCTTCCAGC |
| TBCL18 NR Terminator Fwd | GCTGGAAGATGGCGATTAGGTTCTTGCATGGCCTTTTGATTG |
| TBCL20 DS loxP Fwd | GATCATAACTTCGTATAGCATAC |
| TBCL31 Cre Seq2 Fwd | GCGCTGGAGTTTCAATACCG |
| TBCL32 loxP CNAT Rev V2 | GAACGTGACTCGTTGACGGCAGGTATATTATATCACATACATCG |
| TBCL33 NR Term BB Fwd V2 | TACATGGAGTGCGAAGATC |
| TBCL34 NR Term Rev V2 | GATCTTCGCACTCCATGTAGGAATCTGACATGGAAGTTTG |
| TBF1 Piece 1 Fwd | CAAAATATAATACAAACTATAAGATGTTATC |
| TBF3 Piece 2 Fwd | GTTCGTTCACTCATGGAAAATAG |
| TBF4 Piece 2 Rev | CGCGCCCCTGCAGGAC |
| TBF5 | GTACGGTCAGAAGATTGGACATCTTGAAGACAGTATCACCCAG |
| TBF6 | TTCTTATTCATAGTGTACATGGAGTGCCGCGGTTCCGAATTG |
| ShBle for CreLox insertion (1 F) | CCTCAATCAAGTCTATTTAAATTTCAAAATGGCTAAGTTGACTTCTGCTGT |
| ShBle for CreLox insertion (372 R) | GGATCACGCGGCCGCTTAATCCTGCTCCTCAGCCACG |
| TB61 Gapdh BB Rev | TTTGAAATTTAAATAGACTTGATTGAGGTAG |
| TB62 Gapdh BB Fwd | TAAGCGGCCGCGTGATC |
| LhcA7SeqF | CAGGAAACGAGGCTGAGGTC |
| LhcbM2 Fwd | TTGATGGGACCCACCTTTGG |
| LhcbM2 Rev | CCCTGTGACAGCTTCCAGTG |
| TBL 3 LhcbM139 Fwd | CAATCCAATCAGCATCATAAAATGC |
| TBL 4 LhcbM139 Rev | AGAATTGGATCCATTCGATGCATC |
| TBL 5 LhcbM140 Fwd | CACTATCCACAGGAATGCAGTC |
| TBL 6 LhcbM140 Rev | AAGACATCATTAAATGGAACACTGC |
| TBL 7 LhcbM295 Fwd | CCAGTCAAATTTCGACGATCTATTC |
| TBL 8 LhcbM295 Rev | CTGAAGGATATTCAAGGAAACTATAC |
| TBL 9 LhcbM471 Fwd | CAGAAAATAATTGAATTATTTCAACAATATAATATTTGC |
| TBL 10 LhcbM471 Rev | TTCAATCTAGAATAGCAATGCCGTC |
| TBL 11 Lhca7 591 Fwd | GCTCACACAGACAAGTTCGG |
| TBL 12 Lhca7 591 Rev | ATGTAATCATTCGAATACGTTGATGAG |
| TBL9V2 LhcbM471 M3 Fwd V2 | TCTTCCAGATAACCGCCGTG |
